# Aqueous-Phase Degradation Mechanism of Parabens, Emerging Contaminants, by Peroxynitrite

**DOI:** 10.1021/acsestwater.5c00362

**Published:** 2025-06-18

**Authors:** Clara I. Alcolado, Elena Jiménez, Luis García-Río, Francisco J. Poblete

**Affiliations:** † Universidad de Castilla-La Mancha (UCLM), Facultad de Ciencias y Tecnologías Químicas, Departamento de Química Física, Avda. Camilo José Cela 1B, 13071 Ciudad Real, Spain; ‡ Instituto de Investigación en Combustión y Contaminación Atmosférica, UCLM, Camino de Moledores s/n, 13071 Ciudad Real, Spain; § Universidad de Santiago de Compostela, Facultad de Química, Departamento de Química Física, Avda. Das Ciencias s/n, 15701 Santiago de Compostela, Spain

**Keywords:** pseudo-persistent pollutants, oxidation reaction, environmental chemistry, kinetic study, QSAR

## Abstract

Advances in science and technology have improved the quality of life but have also contributed to environmental pollution through additives like parabens, commonly used as preservatives in personal care products. This study investigates the degradation of a series of parabens (methylparaben, ethylparaben, propylparaben, and butylparaben) in aqueous-phase solution initiated by peroxynitrous acid (HOONO), an oxidant present in environmental waters. A stopped flow system was employed to follow this oxidation reaction. A reaction mechanism, considering experimental variables, such as pH, temperature, ionic strength, radical scavengers, and reaction products detected, was proposed. This mechanism involves two competitive pathways: a radical attack producing hydroquinone and quinone and a nucleophilic attack by HOONO yielding *p*-hydroxybenzoic acid, a less ecotoxic compound than parabens. The acidic equilibrium constants of the carbonyl group of parabens and the rate constants for the nucleophilic attack of HOONO on parabens were experimentally determined. Additionally, a structure–reactivity correlation analysis (QSAR) through the Taft equation was applied for the determined equilibrium and nucleophilic attack rate constants. The derived QSAR can be applied to other water-soluble parabens.

## Graphical abstract

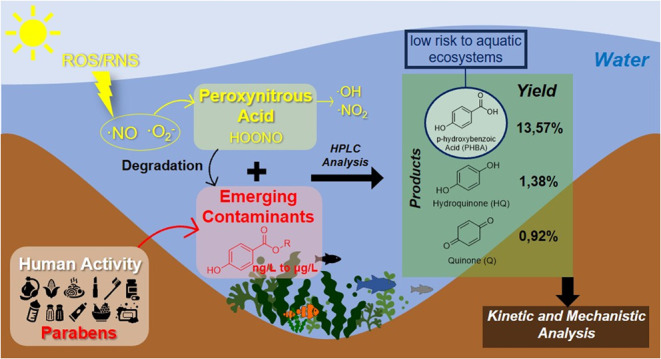

## Introduction

In the European Union (EU), there are approximately 100,000 surface water bodies, including streams, rivers, lakes, wetlands, and reservoirs. However, only 40% of these water bodies are in good ecological and chemical conditions. EU aims for all surface waters to meet standards for biological, hydromorphological, and chemical quality, and to reduce harmful pollutants.^1^ In October 2022, the European Commission adopted a proposal to revise the list of priority substances in surface water, which included the addition of 25 new substances to the priority list. These substances include per- and polyfluoroalkyl substances (PFAS), pesticides, bisphenol A, personal care products (PCPs), pharmaceuticals, and silver, all of which pose significant risks to the environment and human health.^2–4^ These contaminants of emerging concern (CECs) can be bioaccumulated, posing risks to aquatic life and, inevitably, by altering the food chain, to humans.^5,6^ Particularly, the use, especially as preservatives in PCPs, of parabens has increased over time, but the concern on their effect on human health in the EU started in 2004, when their endocrine-disrupting potential and their link to breast cancer were advised.^7,8^ Parabens such as methylparaben (MP), ethylparaben (EP), propylparaben (PP), and butylparaben (BP) have not only been identified in PCPs,^9^ but also in pharmaceuticals^10^ and food,^11^ but at much lower levels.

Due to their widespread use, parabens are found in all types of environmental water, such as drinking and surface waters,^12,13^ wastewater,^14^ and seawater.^15^ The continuous release of parabens has led them to be termed “pseudo-persistent contaminants”,^16^ with variable persistence depending on the environmental conditions,^17,18^ which has led to great efforts to develop efficient removal processes. In aquatic environments, parabens can be degraded by reactive transient species, like nitric oxide (^•^NO), nitrogen dioxide (NO_2_^•^), superoxide anion (^•^O_2_^–^), or hydroxyl radicals (^•^OH), formed from reactive oxygen/nitrogen species. Due to the presence of these radical reactive species in environmental waters, peroxynitrous acid (HOONO) and peroxynitrite anion (ONOO^–^) can be formed. The formation of ONOO^–^ anion from the ^•^NO + O_2_^–^ reaction occurs mainly in animal tissues.^19^ In water, the solubility of NO at 25 °C is low (1.47 × 10^–3^ M at pH 2 and *I* = 0.1 M),^20^ but like the reaction is diffusion-controlled with a rate coefficient *k*_formation_ = 4.3 × 10^9^ M^–1^ s^–1^,^21^ this reaction can be produced in water bodies.^22^ Although progress has been made in the detection and characterization of HOONO, its presence and quantification in natural aquatic systems remain poorly understood.^23^ For example, the work by Adesina et al.^24^ presents a fluorescence-based analytical method that detects photochemically generated HOONO in natural seawater. When this method was applied to 13 seawater samples from the Seto Inland Sea in Japan, they reported concentrations of ONOO^–^ ranging from 0.98 × 10^–11^ to 6.11 × 10^–11^ M, with corresponding photoproduction rates of (0.06–5.13) × 10^–9^ M s^–1^ and lifetimes between 0.01 and 0.16 s. The fact that both peroxynitrite and parabens may be present in aquatic systems highlights the importance of understanding their reactivities in these environments.

As far as we know, several studies, including a computational and a review article, have reported on paraben degradation via OH radicals.^25–28^ For example, Gao et al.^28^ reported that the degradation of MP, EP, PP, and dibutyl paraben initiated by ^•^OH radicals yields the products of the ^•^OH addition route, which is influenced by the length of the alkyl chain, i.e., shorter chains favor the degradation route, while longer chains favor the hydrogen abstraction. The conclusion of the work of Gao et al.^28^ was that the resulting ^•^OH addition products presented higher toxicity to algae and fish than the starting parabens. No kinetic data are available for reactions of parabens with ^•^NO, NO_2_^•^, ^•^O_2_^–^, or HOONO. As HOONO degrades a wide range of organic contaminants^29,30^ and it can be found in aquatic environments together with parabens, the knowledge of the reaction kinetics and degradation mechanism in aqueous solution would be of great help to know how these molecules are degraded, and which reaction products are formed.

Thus, this study focuses, for the first time, on the reaction kinetics and degradation mechanism of MP, EP, PP, and BP initiated by highly reactive HOONO in aqueous solution. Finally, the quantitative structure–activity relationship (QSAR) for the rate constants of the nucleophilic attack reaction and the equilibrium constant for the protonated ester determined in this work was analyzed by using the Taft equation. From this study, the Taft parameters were determined, which allow the results obtained to be extrapolated to other water-soluble parabens.

## Experimental Methods

### Reagents and Chemicals

The synthesis of HOONO was adapted from the method by Leis,^31^ and it was selected for the high yield (almost 100%) and stability of the obtained peroxynitrous acid. The synthesis involves two steps:^29^ the formation of the precursor 2-ethoxyethylnitrite and the subsequent preparation of ONOO^–^ by adding H_2_O_2_. ONOO^–^ is formed by mixing 0.2 mL of 2-ethoxyethylnitrite with 15 mL of 0.109 M H_2_O_2_ and 15 mL of 2 M sodium hydroxide (NaOH). The resulting solution of HOONO/ONOO^–^ is stored at −18 °C, and its concentration was determined daily by spectrophotometry at 302 nm.

All parabens (MP, EP, PP, and BP) are AR grade and purchased from Sigma-Aldrich. And the HPLC standards hydroquinone (HQ), *p*-hydroxybenzoic acid (PHBA), and quinone (Q) were purchased from Sigma-Aldrich with a purity of ≥99%, whereas methyl 3,4-dihydroxybenzoate, methyl 2,4-dihydroxybenzoate, methyl 4-hydroxy-3-nitrobenzoate, and methyl 4-hydroxy-2-nitrobenzoate were obtained from Aaron Chemicals with a purity of 98%. All solutions were prepared with double-distilled water, except in the synthesis of peroxynitrous acid, where ultrapure water from a Milli-Q system (Millipore, Bedford, MA) was employed.

### Stopped Flow Experiments

The results were obtained using the experimental system Stopped Flow SX-20MV spectrometer from Applied Photophysics, equipped with an anaerobic kit (this system prevents external gases from entering the stopped flow syringes), and chosen to mix two solutions (A and B) in a 1:1 mixing ratio. Both solutions were previously bubbled with argon for 15 min to ensure the removal of dissolved CO_2_ and O_2_. Solution A contains the paraben ((1.00–8.00) × 10^–5^ M), Na_2_SO_4_ used for adjusting the ionic strength (*I* = (0.025–0.3) M), and H_2_SO_4_ to vary the pH (1–4). Although the use of the method of the initial rates makes pH changes irrelevant, this parameter was checked at the beginning and end of all kinetic experiments with a pH meter (Crison, model micropH 2000). No significant changes in pH were observed. Solution B contains HOONO ((4.00–12.00) × 10^–5^ M) at a selected ionic strength.

The kinetic experiments, carried out as a function of the temperature (10–30 °C), started with the mixing of both solutions. For that purpose, two synchronized syringes propel solutions A and B into a mixing chamber in a millisecond time scale. The reaction mixture then flows through a short tube to the observation chamber to minimize the dead time. A third syringe triggers an electronic signal that stops the flow and activates a spectrophotometer to monitor the reaction by measuring the absorbance changes over time.

The paraben was chosen as the control chemical species. This species was monitored during the kinetic experiments by UV spectroscopy at the maximum absorption wavelength, 255 nm, and its concentration was determined by using the Beer–Lambert law.

### Analytical Methods

The detection of methylparaben and its reaction products (hydroquinone, quinone, and *p*-hydroxybenzoic acid) was performed using an HPLC (Agilent 1265 Infinity II) equipped with a UV photodiode array detector (Varian ProStar 335) of 1024 elements, which can measure the total absorbance at wavelengths from 190 to 950 nm, providing complete spectral and chromatographic peak processing. The HPLC parameters for the products determination included an injection volume of 10 μL. The mobile phase consisted of a mixture of water and acetonitrile in a gradient from 90 to 10% V/V and was pumped at a flow rate of 0.5 mL/min through a Microsorb-MV 100, C18, 100 Å, 4.6 mm × 250 mm, 5 μm (Agilent, Santa Clara).

The methylparaben and the reaction products were detected by UV spectroscopy at different absorption wavelengths as summarized in Table 1.

**Table 1:** Detection Wavelength in the HPLC System

reference species	λ (nm)
MP	255
HQ	290
Q	245
PHBA	255
methyl 3,4-dihydroxybenzoate	260
methyl 2,4-dihydroxybenzoate	254
methyl 4-hydroxy-3-nitrobenzoate	240
methyl 4-hydroxy-2-nitrobenzoate	235

The identification and quantification process first involved the introduction of the selected standards of known concentration into the HPLC for their correct identification later. Then, a reaction sample was prepared at a pH of 2. In the sample, the concentrations of MP and HOONO were kept constant at 8.00 × 10^–5^ and 1.20 × 10^–4^ M, respectively. The ionic strength was fixed at 0.1 M in these experiments. Then, the sample was introduced into the HPLC at a pressure of 39.18 bar and a temperature of 30 °C and reacted for 15 min to ensure a proper concentration of the oxidation products to be detected.

The rest of parabens (EP, PP, BP) would present the same behavior. This fact was verified by ^1^H NMR.

### Data Analysis

The analysis of the experimental data was carried out by the initial rate method, which eliminates problems such as interference from the reaction products and the presence of side reactions. Absorbance–time data were collected and fitted to a four-degree polynomial ([Disp-formula eq1]),^29^ with statistical analysis using the Origin2019b program. The advantage of using a fourth-degree polynomial fit to the experimental data and subsequently deriving the resulting equation is that all data are considered in the fitting (500 points in our study). It ensures a better representation of the experimental data across the entire range, allows for the analytical derivation of the rate equation, and enables a more precise calculation of the initial rate. The quality of the polynomial fit for the same example indicates a much stronger correlation and reduces the potential for bias introduced by subjective point selection. The polynomial model was used only to extract the initial slope analytically and not to interpret mechanistic behavior, thus minimizing the risk of overinterpretation.

E1 $$A=a+\mathrm{bt}+ct^{2}+dt^{3}+et^{4}$$

where *A* is the absorbance of parabens at 255 nm, directly related with its concentration via the Lambert–Beer’s law, and *t* is the reaction time. The initial reaction rate (v_0_) ([Disp-formula eq2]) can be obtained, then, by deriving eq 1 with respect to reaction time

E2 $$v_{0}=-{\left(\frac{d\left[Paraben\right]}{dt}\right)}_{0}=-\frac{1}{\varepsilon \cdot 1}{\left(\frac{dA}{dt}\right)}_{0}=-\frac{1}{\varepsilon \cdot 1}\cdot b$$

where *b* is the coefficient of the first-order term in eq 1.

Under our experimental conditions, the validity of the initial reaction rate method was checked by registering a complete time profile of the absorbance of paraben in the presence of HOONO. An example of the absorbance versus time plot is given in the Supporting Information (Figure S1 and Table S1). The best fit of the observed profile was a fourth-grade power equation. The first derivative of the fitted equation allows evaluation of the initial reaction rate at *t* = 0.

To ensure the accuracy and reproducibility of this study, the initial reaction rate data reported in this work are the mean values of five independent experiments performed under the same experimental conditions. The standard error (SE) was calculated using Origin2019b software scaled by the square root of the reduced χ-square. Unlike standard deviation, which measures variability within a sample, SE quantifies the uncertainty in estimated parameters across samples, making it more appropriate for this study.

## Results and Discussion

In this section, the analysis of the effect of the reagent concentration (paraben and HOONO) on the initial reaction rate to determine the experimental rate equation is presented. Subsequently, the effects of pH, ionic strength, and temperature on the reaction rate are also examined. Finally, the impact of free radicals and the decomposition products of HOONO (nitrates and nitrites will be investigated to propose a reaction mechanism).

To analyze the influence of the concentration of parabens or peroxynitrous acid on the initial reaction rate, a series of experiments have been carried out where the pH, ionic strength, and temperature were kept constant and the concentration of parabens and HOONO was varied. Figure 1A shows the variation of the paraben concentration vs the initial reaction rate at a fixed concentration of HOONO. As it can be seen, v_0_ increases nonlinearly with increasing paraben concentration, implying that the reaction order with respect to paraben is complex. The values of v_0_, however, tend to reach a maximum value, i.e., a zero-reaction order, with respect to the paraben. Figure 1B shows a linear variation of v_0_ with the concentration of peroxynitrous acid at a constant paraben concentration, which implies a first reaction order with respect to HOONO. Both figures show that the initial reaction rate increases as the length of the hydrocarbon chain decreases. However, no significant increase has been observed from PP to BP. The interpretation of this phenomenon is discussed later in detail at the end of the Results and Discussion section in terms of the Taft structure–reactivity correlation.

**Figure 1:**
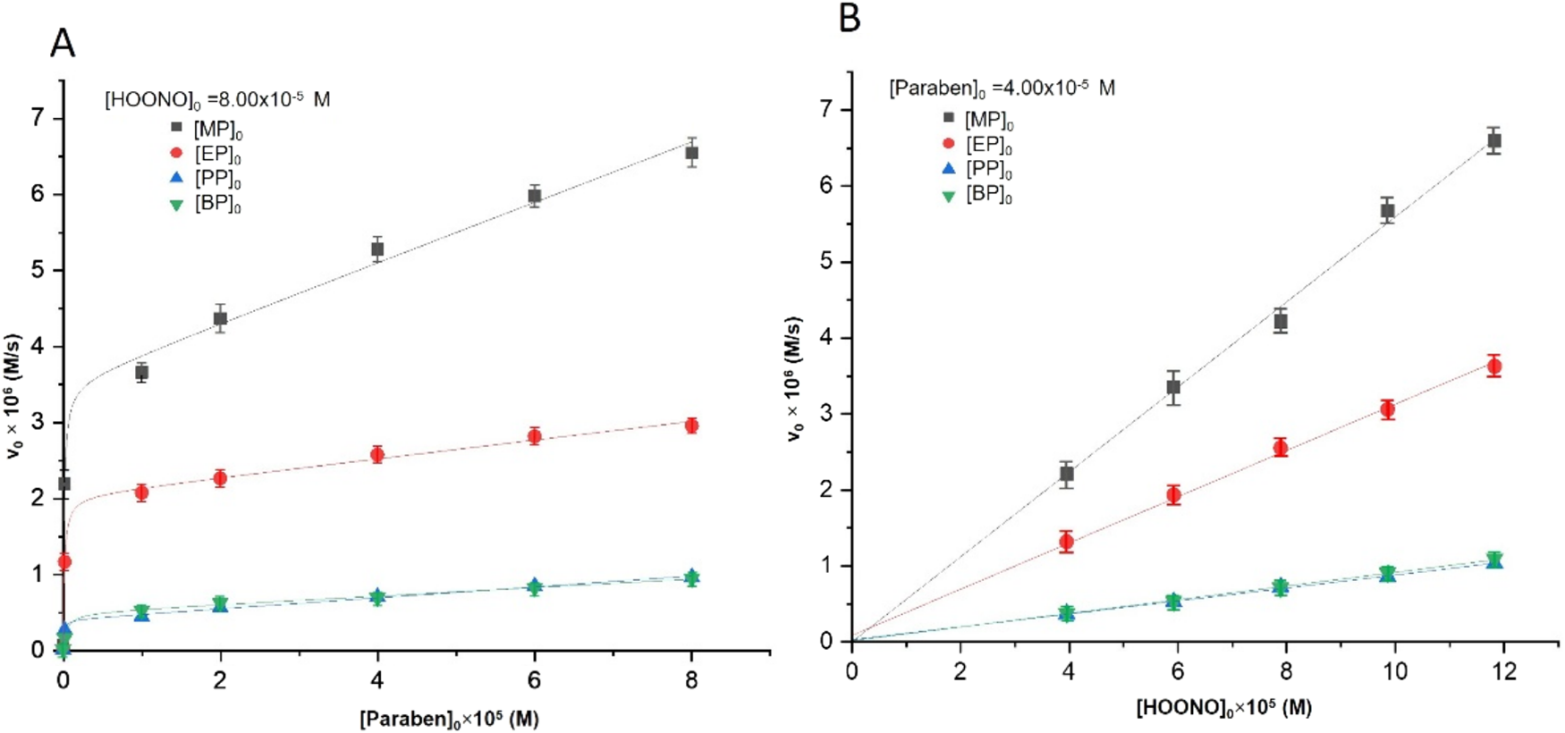
Influence of the parabens (A) and peroxynitrous acid (B) concentrations on the initial reaction rate (* [Paraben] where MP (methylparaben), EP (ethylparaben), PP (propylparaben), BP (butylparaben)). Experimental conditions: *I* = 0.1 M; *T* = 25 °C; pH = 2.

The experimental data for each paraben are in the Supporting Information, Table S2 and Figures S2–S5. The best mathematical fit of the experimental data was obtained using Origin2019b software, after testing multiple fitting equations, the most accurate representation within the best statistical metrics in eqs 3 and 4. From Figure 1A, the rational eq 3 was obtained, while Figure 1B fits a straight line of eq 4.

E3 $$v_{0}=\frac{A{\left[\mathrm{Paraben}\right]}_{0}+B{\left[\mathrm{Paraben}\right]}_{0}^{2}}{1+C{\left[\mathrm{Paraben}\right]}_{0}}$$

E4 $$v_{0}=D{\left[\mathrm{HOONO}\right]}_{0}$$

Table S7 shows the *A*, *B*, *C*, and *D* parameters obtained for the mathematical fitting to eqs 3 and 4. Parameters *A*, *B*, and *C* are dependent on [HOONO]_0_ and pH, while *D* depends on [Paraben]_0_ and pH.

The influence of pH on the initial reaction rate was conducted only under acidic conditions, since at a basic pH of 8, which is above p*K*_a_ of the molecule, there is a bathochromic shift in the absorption maximum of paraben to 297 nm due to the deprotonation of the paraben and subsequent formation of the anion favoring increased absorption and mobility of π electrons.^17^ This shift causes an overlap with the absorption band of peroxynitrous acid at 302 nm. Thus, in order to analyze the effect of pH on the initial reaction rate, a series of experiments between pH = 1 and 4 were carried out in which paraben (4.00 × 10^–5^ M) and peroxynitrous acid (8.00 × 10^–5^ M) concentrations, ionic strength (0.1 M), and temperature (25 °C) were kept constant.

In Table 2, it is observed that v_0_ increases with an increasing proton concentration. This increase is not linear. A process governed by specific acid catalysis, the theoretical slope of a log v_0_ vs pH plot would be −1. In our case, in Figure 2, the observed slopes deviate significantly from this ideal value, indicating that the degradation process of parabens is more complex and cannot be solely attributed to specific acid catalysis.

**Figure 2:**
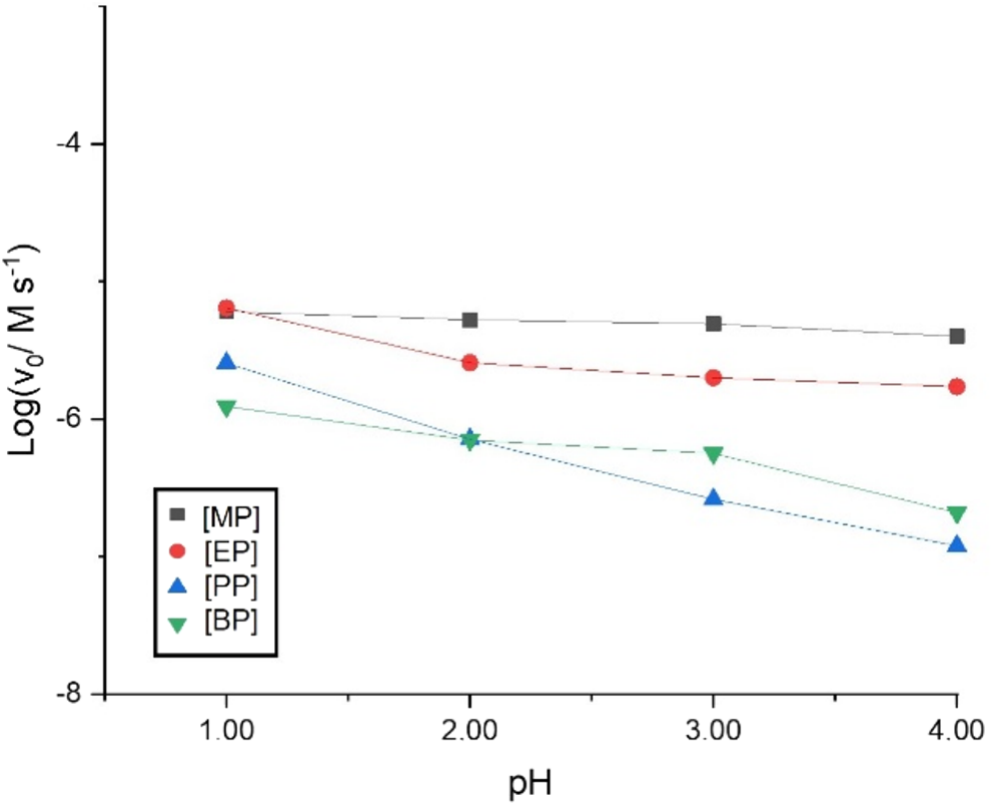
Plot log v_0_ vs pH in acidic media. Experimental conditions: *T* = 25 °C, *I* = 0.1 M, [Paraben]_0_ = 4.00 × 10^–5^ M, [HOONO]_0_ = 8.00 × 10^–5^ M.

**Table 2:** Influence of pH on the Initial Reaction Rate^a^

	v_0_ × 10^7^ (M·s^–1^)			
pH	1	2	3	4
MP	60.3 ± 0.02	52.8 ± 0.01	49.4 ± 0.04	40.0 ± 0.02
EP	64.5 ± 0.01	25.8 ± 0.02	20.1 ± 0.01	17.3 ± 0.02
PP	25.6 ± 0.03	7.14 ± 0.01	2.62 ± 0.02	1.20 ± 0.03
BP	12.4 ± 0.02	7.05 ± 0.03	5.66 ± 0.02	2.09 ± 0.01

a Experimental conditions: [Paraben]_0_ = 4.00 × 10^–5^ M, [HOONO]_0_ = 8.00 × 10^–5^ M, *I* = 0.1 M, *T* = 25 °C.

Figure S6 shows the experimental data as a function of the proton concentration. The best mathematical fit of the experimental data is given in eq 5.

E5 $$v_{0}=\frac{X\left[H^{+}\right]+Y{\left[H^{+}\right]}^{2}}{1+Z\left[H^{+}\right]}$$

where parameters *X*, *Y*, and *Z* are a compendium of different constants, as shown in eq 7.

To study the influence of ionic strength on the reaction rate, a series of experiments were carried out in which the parabens and HOONO concentration, temperature, and pH were kept constant, while the ionic strength was varied by adding the corresponding amount of Na_2_SO_4_. The results are shown in Table S3, where it can be observed that v_0_ is not affected by the variation of the ionic strength in any paraben. This result indicates that a neutral species is involved in the reaction rate-limiting step.

These experiments were performed only to elucidate the reaction mechanism and to understand the behavior of HOONO within the system. As shown in Table S4, the addition to the reaction mixture of the HOONO decomposition products, NO_2_^–^ or NO_3_^–^, does not change the initial reaction rate, implying that the equilibria between HOONO and NO_2_^–^ or NO_3_^–^ does not affect the rate-determining stage.

The formation of radicals in the reaction mixture has been checked using acrylic acid (AA) as an effective radical scavenger.^32^ As depicted in Figure 3 (Table S5), when AA was added to the reaction mixture, the initial reaction rate decreased until stabilization, when [AA]_0_ increases. For example, for an initial concentration of MP of 1 × 10^–5^ M and [HOONO]_0_ = 4 × 10^–5^ M, *T* = 25 °C, pH = 2, and *I* = 0.1 M, v_0_ was 2.22 × 10^–6^ M/s and in the presence of [AA]_0_ = 4 × 10^–6^ M, v_0(AA)_ was 8.72 × 10^–7^ M/s (v_0(AA)_/v_0_ = 0.4). Therefore, radical species exist in the reaction medium. For the four parabens studied, the proportion of the [AA]_0_/[HOONO]_0_ was around 2:1, which implies that the nonradical reaction pathway predominates. Moreover, when the hydrocarbon chain of the paraben grows, the initial reaction rate decreases. This suggests that the rate constants of nonradical processes are lower for parabens with long hydrocarbon chain.

**Figure 3:**
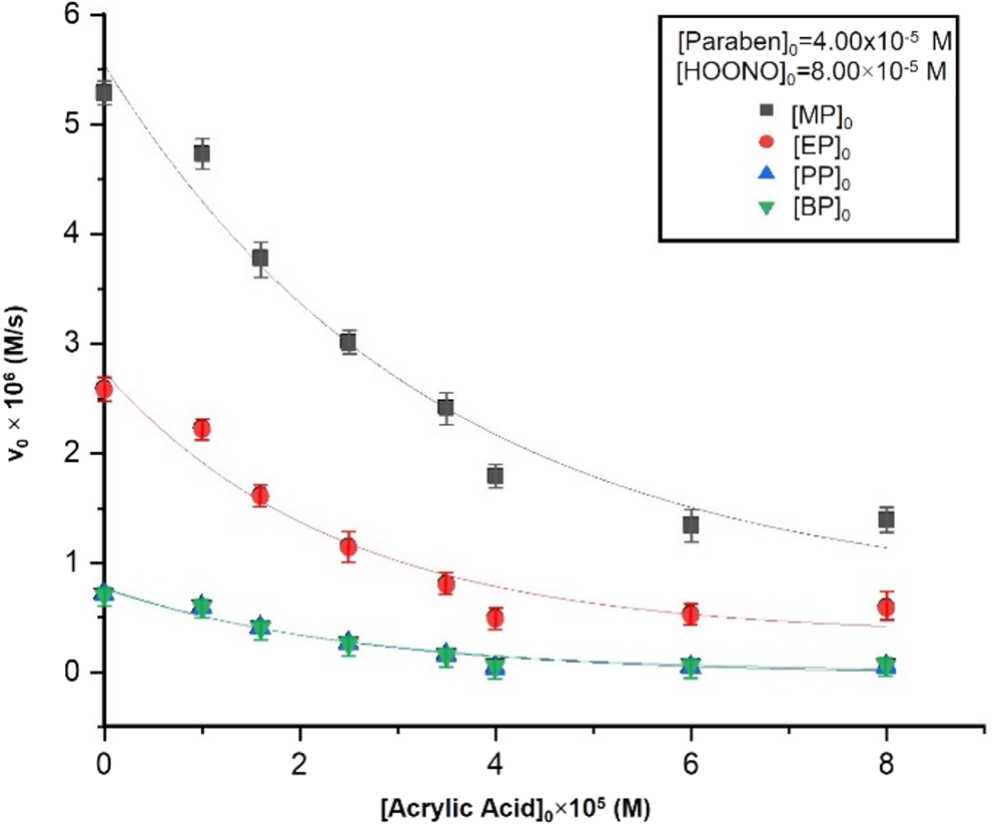
Influence of the concentration of the free radical scavenger (acrylic acid) on the initial reaction rate. Experimental conditions: *T* = 25 °C, pH = 2, *I* = 0.1 M, [Paraben]_0_ = 4.00 × 10^–5^ M, [HOONO]_0_ = 8.00 × 10^–5^ M.

To analyze the effect of temperature on the initial reaction rate, a series of experiments were performed in which the temperature was varied from 10 to 30 °C, maintaining constant the paraben and the peroxynitrous acid concentrations, the ionic strength of the medium, and pH. As can be seen in Table S6, as the temperature increases, the reaction rate also increases.

The products of the reaction between MP and HOONO in acidic medium (pH = 2) were identified by HPLC (see Supporting Information, Figure S9). The identified reaction products were quinone (Q), hydroquinone (HQ), and *p*-hydroxybenzoic acid (PHBA). The molar yield of the reaction products is summarized in Table 3. The identification of the reaction products allows us to propose a reaction mechanism (see next section). For EP, PP, and BP, the formation of the same aromatic products is expected, with PHBA being the main reaction product. Also, the nitro and hydroxy compounds listed in Table 1 (methyl 3,4-dihydroxybenzoate, methyl 2,4-dihydroxybenzoate, methyl 4-hydroxy-3-nitrobenzoate, methyl 4-hydroxy-2-nitrobenzoate) were not detected as reaction products.

**Table 3:** Concentration of the Reaction Products and Molar Yield at 15 min and pH = 2^a^

reaction product	concentration × 10^6^ (M)	yield (%)
*p*-hydroxybenzoic acid (PHBA)	5.43 ± 0.08	13.6 ± 0.12
hydroquinone (HQ)	0.55 ± 0.02	1.38 ± 0.01
quinone (Q)	0.37 ± 0.01	0.92 ± 0.03

a Experimental conditions: [HOONO]_0_ = 1.20 × 10^–4^ M, [Paraben]_0_ = 8.00 × 10^–5^ M, *I* = 0.1M, *t* = 15 min, *T* = 30 °C.

The study of the reaction products was made only at the highest concentration of paraben and HOONO to obtain higher concentrations of these compounds and achieve greater sensitivity and selectivity in the HPLC technique.

The mechanism that justifies all experimental results must consider the decomposition of peroxynitrite as a source of reactive species, such as HOONO and radical species such as ^•^NO_2_ and ^•^OH. The rate constant for the HOONO decomposition was determined by our research group to be *k*_1_ = (1.67 ± 0.01) s^–1^, which provides a lifetime for HOONO of 0.6 s.^33^

The proposed reaction mechanism is presented in Scheme 1. Initially, acid–base equilibria are established to form the active species of peroxynitrite (reaction R1) and paraben (reaction R2). In the proposed mechanism, there are two competitive stages (R5–R9):

- Radical attack to paraben: Oxidation of the paraben is produced by radicals generated from the decomposition of HOONO to yield hydroquinone and quinone (R3, R4).
- Nucleophilic attack of HOONO on the carbonyl carbon of the paraben to generate an adduct (X) (R5), which evolves to form *p*-hydroxybenzoic acid directly (R6) or with a reaction of another paraben (R7).

**Scheme 1:**
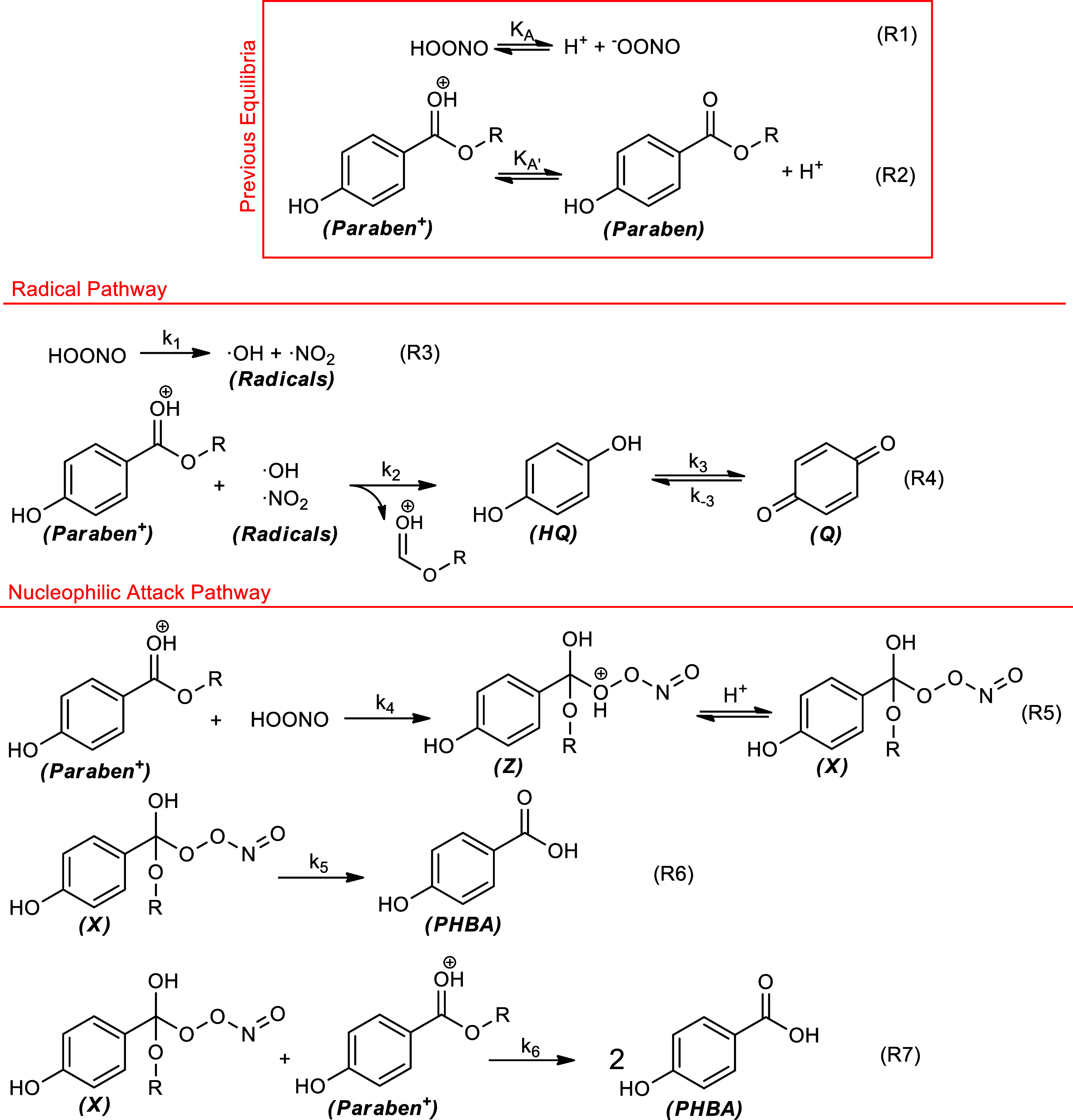
Reaction Mechanism

The theoretical rate equation obtained from the proposed mechanism is given in eq 6.

E6 v0=k2[Paraben+][OH•]+k2[Paraben+][NO2•]+k4[Paraben+][HOONO]+k6[X][Paraben+]

The first two terms in eq 6 correspond to the oxidation of paraben by the radicals formed in peroxynitrite decomposition (R4). As [^•^OH] = [^•^NO_2_] and both radicals present a similar reactivity (close to the diffusion limit), it could be assumed that *k*_2_[Paraben^+^] [^•^OH] = *k*_2_[Paraben^+^] [^•^NO_2_]. The second and third terms represent the nucleophilic attack of HOONO over protonated paraben (Paraben^+^) that increases carbonyl carbon’s electrophilicity, to form (X) (R5–R7). So, taking into account the two terms from the competitive mechanistic pathways, applying the steady state approach to [X] and considering the mass balance equation with respect to peroxynitrous acid, the theoretical rate equation can be written as eq 7. The step-by-step deduction is shown in the Supporting Information.

E7 $$v_{0}=\left\{2k_{1}k_{5}K_{A\prime }^{2}+2k_{1}k_{6}K_{A\prime }{\left[\mathrm{Paraben}\right]}_{0}\left[H^{+}\right]+k_{4}k_{5}K_{A\prime }{\left[\mathrm{Paraben}\right]}_{0}\left[H^{+}\right]+2k_{4}k_{6}{\left[\mathrm{Paraben}\right]}_{0}^{2}{\left[H^{+}\right]}^{2}\right\}/\left\{k_{5}K_{A\prime }^{2}+k_{6}K_{A\prime }{\left[\mathrm{Paraben}\right]}_{0}\left[H^{+}\right]\right\}{\left[\mathrm{HOONO}\right]}_{0}A^{\prime }$$

where *A*′ is

E8 $$A^{\prime }=\left(\frac{\left[H^{+}\right]}{[H^{+}]+K_{A}}\right)$$

To determine for the first time the acid equilibrium constant of paraben *K*_A′_, a Sigman mechanism^34^ for ester hydrolysis is considered (Figure S7). The equilibrium constant between paraben and H^+^ to form protonated paraben, *K*_B_, and the rate constant of the formation of the product from protonated paraben (*k*) can be obtained using the expression that correlates v_0_ with [H^+^] (eq 9).

E9 $$\frac{v_{0}}{{\left[\mathrm{Paraben}\right]}_{0}}=\frac{k\left[H^{+}\right]\left[H_{2}O\right]}{1+K_{B}\left[H^{+}\right]}$$

Since the medium was very acidic (pH = 1–4), acidity functions, *h*_0_ = 10^–H0^ can be used instead of [H^+^].^35^ According to the method described by Beel et al.^36^ and Yates et al.,^37^ v_0_/[Paraben]_0_ was plotted against *h*_0_ [29] and fitted to expression [Disp-formula eq9], with *K*_B_ and *k* listed in Table 4. It can be observed that *k* and K_B_ increase with the alkyl chain of the paraben; the variation of *K*_B_ is as expected since the protonated compound is more stable when alkyl chains increase at acidic pH due to the inductive effects of the hydrocarbon chain.

**Table 4:** Parameters *k* and *K*_B_ of Sigman Mechanism, and Obtained Rate Constants *k*_4_ and *K*_A′_ for Each Paraben^a^

Paraben	*k* × 10^3^ (M^–1^ s^–1^)	*K*_B_ × 10^2^ (M^–1^)	*k*_4_ × 10^–8^ (M^–1^ s^–1^)	*K*_A′_ (M)
MP	0.13 ± 0.01	0.81 ± 0.04	38.7 ± 0.01	123.15 ± 6.06
EP	0.75 ± 0.02	1.94 ± 0.08	6.63 ± 0.02	51.49 ± 2.12
PP	0.86 ± 0.03	3.52 ± 0.09	3.35 ± 0.01	28.44 ± 0.73
BP	2.77 ± 0.01	7.23 ± 0.09	1.03 ± 0.03	13.82 ± 0.17

a Experimental conditions: [Paraben]_0_ = 4.00 × 10^–5^ M; [HOONO]_0_ = 8.00 × 10^–5^ M, *T* = 25 °C.

Knowing *K*_A′_, that is *K*_A′_ = 1/*K*_B_, the rate constant for the nucleophilic attack of peroxynitrous acid to the carbonyl carbon of paraben to form the adduct (X) (*k*_4_) has been determined in this work for the first time. As Table 4 shows, the rate constant *k*_4_ decreases as the hydrocarbon chain attached to the oxygen increases in size, which indicates a stabilization of the charge as the chain increases. For all parabens, the equilibrium (R4) is shifted to the protonated form of the paraben, and it strongly decreases with the hydrocarbon chain of the paraben. A low value of *K*_A′_ implies a higher concentration of the protonated species of the paraben, favoring the nucleophilic attack, which also promotes the formation of PHBA, a product less harmful than hydroquinone derivatives. This is observed for BP. In contrast, the higher *k*_4_, the greater the formation of PHBA, as observed for MP. This conclusion is in agreement with the results obtained in the free radical test and the conclusions drawn from Figure 3.

Gao et al.^28^ reported the theoretical rate constants for the radical pathway of neutral parabens (MP, EP, PP, and BP) with ^•^OH at room temperature (298 K). These authors observed that the rate constant for these reactions slightly increases with the hydrocarbon length of paraben. The rate constants are on the order of 10^9^ and 10^8^ M^–1^ s^–1^, similar to those determined in the present work for the protonated parabens (*k*_4_), implying that they are controlled by the diffusion of ^•^OH and HOONO. No rate constants for the reaction of parabens with ^•^NO_2_ have been found in the literature.

Quantitative structure–activity relationship (QSAR) analysis allows us to estimate the rate constant or the equilibrium constant for other water-soluble parabens like isopropylparaben, isobutylparaben, benzylparaben, phenylparaben, pentylparaben, octylparaben, etc. In addition, QSAR helps in the choice of the necessary amount of oxidant needed for their degradation. The correlation between the structure of the studied parabens and their reactivity toward peroxynitrous acid can be obtained by applying the Taft eq 10.^38^ Moreover, the Taft relationship can be applied to *K*_A′_.

E10 $$\ln\frac{k_{R}}{k_{{\mathrm{CH}}_{3}}}=\rho^{*}\cdot \sigma_{R}^{*}+\delta \cdot E_{S}$$

where:

*k*_R_ is the rate constant of HOONO with the aliphatic compound with the substituent R.

*k*_CH_3__ is the rate constant of HOONO with the aliphatic compound with the substituent CH_3_.

ρ* indicates the susceptibility of the reaction to the electronic effects of the substituents.

σ_R_* is the constant measuring the electronic effect of the substituent.

δ is the susceptibility of the reaction to the steric effects of the substituents.

*E*_s_ is the constant measuring the steric effect of the substituent.

Table S12 summarizes the values of ln *k*_4_ and ln *K*_A′_, and the steric and electronic constants for each paraben.^38–40^ The Taft equation can be rearranged, as shown in eq 11.

E11 $${\ln k}_{R}={\ln k}_{{\mathrm{CH}}_{3}}+\rho^{*}\cdot \sigma_{R}^{*}+\delta \cdot E_{S}$$

From this expression, two linear fits can be obtained. If the natural logarithm of *k*_4_ and *K*_A′_ is plotted against the steric constants of each paraben, it is possible to obtain the susceptibility of the reaction to the steric effects of the substituents Figure S8A. On the other hand, if the natural logarithm of *k*_4_ and *K*_A′_ is plotted against the inductive constants of each paraben, it is possible to qualitatively determine the susceptibility of the reaction to the electronic effects of the substituents Figure S8B.

From the slopes of the previously represented lines (Figure S8), it is possible to determine the qualitative parameters ρ and δ for the Taft equation, and the resulting expressions are given by eqs 12 for *k*_4_ and 13 for *K*_A′_.

E12 $$\ln\left(k_{4}/M^{-1}\;s^{-1}\right)=26.06+15.83\cdot \sigma_{R}^{*}+3.17\cdot E_{S},\left(r^{2}=0.9342\right)$$

E13 $$\ln\left(K_{A\prime }/M\right)=8.18+7.09\cdot \sigma_{R}^{*}+2.69\cdot E_{S},\left(r^{2}=0.9446\right)$$

When the absolute value of the steric factor increases, *k*_4_ and *K*_A′_ decrease; theoretically, steric effects are diminished in the transition state.^41^ When ρ* > 0, the reaction accumulates negative charge in the (Z) complex, and therefore, the reaction is favored by electron attractor substituents.

In Table 5, *k*_4_ and *K*_A′_ derived from eqs 12 and 13, respectively, are summarized for a series of parabens. These predictions have to be taken cautiously as they are subject to the uncertainties in the experimental data of Taft et al.^38^ and Pal’m et al.^39^ Parabens with large hydrocarbon chains or aromatic substituents, such as benzylparaben and phenylparaben, show higher *k*_4_ values, suggesting higher nucleophilic attack, probably due to a greater charge stabilization of the (Z) complex. Taking this into account, when the protonated paraben is stabilized, a lower acidity constant *K*_A′_ is observed. This fact could indicate that the aromatic parabens mainly yield PHBA.

**Table 5:** Estimated *k*_4_ and *K*_A′_ for Other Parabens

Paraben	*k*_4_ × 10^–8^ (M^–1^ s^–1^)	*K*_A′_ (M)
isopropylparaben	0.45 ± 0.01	5.65 ± 0.62
isobutylparaben	0.034 ± 0.01	1.47 ± 0.04
benzylparaben	396.7 ± 93.8	64.98 ± 13.2
phenylparaben	152.1 ± 3.16	5.33 ± 1.90
pentylparaben	1.55 ± 0.10	9.29 ± 1.79
octylparaben	1.97 ± 0.17	10.81 ± 2.41

## Conclusions

The present study demonstrates that HOONO efficiently degrades the investigated alkylparabens in acidic aqueous solution, primarily through the nucleophilic attack on the carbonyl carbon of the paraben, producing *p*-hydroxybenzoic acid (PHBA). The rate constants for the nucleophilic attack of HOONO on some water-soluble parabens with long hydrocarbon and aromatic chains (in their protonated form) were estimated from the QSAR analysis presented in this work, which allowed us to estimate. This reaction has been concluded to be very slow for isopropylparaben, isobutylparaben, pentylparaben, and octylparaben. In contrast, for benzylparaben and phenylparaben, the estimated rate constant for the nucleophilic attack of HOONO largely increases with respect to alkylparabens due to the conjugative effects that stabilize the charge of reaction intermediates.

Although small amounts of ONOO^–^ are found in seawater, slight variations in pH can increase the concentration of HOONO, a highly reactive species that reacts immediately upon formation. In the present study, acidic pH conditions (1–4) were used to investigate the mechanism of the reaction of HOONO with parabens. Under these conditions, HOONO is present at the 99.9% level.

From the environmental point of view, as the pH of environmental waters is basic (around pH = 8), the degradation products of parabens are expected to be the same as under acidic conditions.^17^ Indeed, in the present work, the same degradation products, including PHBA, were observed at pH 11 by HPLC analysis, supporting the environmental relevance of the proposed mechanism. As PHBA is expected to be the main degradation product, it is considered a low priority for further environmental control measures^42^ because of its low toxicity in aquatic organisms, which is supported by the No Observed Effect Concentration values for algae (32.0 mg/L) and fish (66.5 mg/L). As the toxicity of parabens increases with increasing the hydrocarbon chain length,^43^ the degradation of other water-soluble ones with long hydrocarbon chain or an aromatic chain would reduce even more their effect on the quality of environmental water.

## Supplementary Material



## Notes

Detailed description of the kinetic study, deduction of the theoretical rate equation, determinations of *K*_A′_ and *k*_4_, and additional graphs and data results (Tables S1–S12 and Figures S1–S9) (PDF)
