# Supplementary material for: Aqueous-Phase Degradation Mechanism of Parabens, Emerging Contaminants, by Peroxynitrite
Source: ACS ES T Water. 2025 Jun 18;5(7):4147–55. doi: 10.1021/acsestwater.5c00362 (PMC12262489; doi:10.1021/acsestwater.5c00362)

SUPPORTING INFORMATION

Aqueous-Phase Degradation Mechanism of Parabens, Emerging Contaminants, by Peroxynitrite

Clara I. Alcolado[a,b], Elena Jiménez[a,b], Luis García-Río [c], and Francisco J. Poblete [a,b,*]

[a] Universidad de Castilla-La Mancha (UCLM), Facultad de Ciencias y Tecnologías Químicas, Departamento de Química Física, Avda. Camilo José Cela 1B, 13071, Ciudad Real (Spain).
[b] Instituto de Investigación en Combustión y Contaminación Atmosférica, UCLM, Camino de Moledores s/n, 13071, Ciudad Real (Spain).
[c] Universidad de Santiago de Compostela, Facultad de Química, Departamento de Química Física, Avda. Das Ciencias s / n. 15701, Santiago de Compostela (Spain).

Figure S1. Example of the absorbance temporal decay of methylparaben due to the oxidation reaction initiated by peroxynitrite. Experimental conditions: $[MP]_0$=1.00×10^{-5}M; $[HOONO]_0$=4.00×10^{-5}M; pH=2; I=0.1 M; T=25ºC 3

Table S1. Fitting parameters of the decay shown in Figure S1 for MP. Experimental Conditions: $[MP]_0$=1.00×10^{-5}M; $[HOONO]_0$=4.00×10^{-5}M; pH=2; I=0.1 M; T=25ºC 3

Table S2. Initial rates for the oxidation of the investigated parabens by peroxynitrite. Experimental conditions: pH=2, I=0.1M; T=25 ºC. 4

Figure S2. Influence of $[MP]_0$ (left) and $[HOONO]_0$ (right) upon the initial rate (v_0). Experimental conditions: pH 2, I=0.1M and T=25ºC 5

Figure S3. Influence of $[EP]_0$ (left) and $[HOONO]_0$ (right) upon the initial rate (v_0). Experimental conditions: pH 2, I=0.1M and T=25ºC 5

Figure S4. Influence of $[PP]_0$ (left) and $[HOONO]_0$ (right) upon the initial rate (v_0). Experimental conditions: pH 2, I=0.1M and T=25ºC 5

Figure S5. Influence of $[BP]_0$ (left) and $[HOONO]_0$ (right) upon the initial rate (v_0). Experimental conditions: pH 2, I=0.1M and T=25ºC 6

Figure S6. Influence of $[H^+]$ upon the initial rate (v_0) for all the parabens. Experimental conditions: $[Paraben]_0$=4.00×10^{-5} M, $[HOONO]_0$=8.00×10^{-5}M, I=0.1M, Tª=25ºC. 6

Table S3: Initial velocity values obtained at different ionic strength values $[Paraben]_0$=4.00×10^{-5} M, $[HOONO]_0$=8.00×10^{-5}M, pH = 2, T=25ºC. 6

Table S4. Influence of the addition of nitrates and nitrites on the initial reaction rate. Experimental conditions: $[HOONO]_0$ = 8.00×10^{-5} M, $[MP]_0$ = 4.00×10^{-5} M, I= 0.1M, pH = 2, T= 25ºC. 7

Table S5. Influence of free radical Scavenger (Acrylic Acid) on the initial reaction rate. Experimental conditions: $[HOONO]_0$ = 8.00×10^{-5} M, $[Paraben]_0$ = 4.00×10^{-5} M, I= 0.1M, pH = 2, T= 25ºC..7

Table S6. Influence of temperature on the initial rate Experimental conditions: $[HOONO]_0$ = 8.00×10^{-5} M, $[Paraben]_0$ = 4.00×10^{-5} M, I= 0.1M, pH = 2. 7

Deduction of the theoretical rate equation according with Scheme 1 of the article. 7

Theoretical rate equation 7

Table S7. Values of the parameters A, B, C and D form the adjust of the representation of initial rate vs substrate or oxidant. Experimental Conditions: T=25°C, pH=2, I=0.1M.8

Table S8. Parameters A, B and C obtained from the fit of the oxidant concentration versus A, B and C parameters from Tables S7. Experimental conditions: I=0.1 M; T=25ºC; pH=2...10

$K_{A'}$ determination...10

Table S9. Initial rate of the different parabens at different pH. Experimental conditions: $[Paraben]_0$=4.00×10^{-5} M; $[HOONO]_0$=8.00×10^{-5}M, T=25ºC. 10

Table S10. Initial rate of the different parabens and h_0. Experimental conditions: $[Paraben]_0$=4.00×10^{-5} M; $[HOONO]_0$=8.00×10^{-5}M, T=25ºC. 11

Figure S7. Singman mechanism for ester hydrolysis 11

Table S11. Parameters k and K_B from the representation of table S10. Experimental conditions: $[Paraben]_0=4.00\times10^{-5}$ M; $[HOONO]_0=8.00\times10^{-5}$M, T=25ºC. 11

k_4 determination 11

Figure S8. Linear fits of natural logarithm of k_4 and $K_{A'}$ plotted against the steric constant (A) and the inductive constant (B) 12

Table S12. Summarizes the values of (ln k_4), (ln $K_{A'}$) and steric and electronic constants for each paraben. 12

Figure S9. HPLC analysis spectra for acid media. Experimental conditions: $[Paraben]_0=8.00\times10^{-5}$ M; $[HOONO]_0=12.00\times10^{-5}$M, T=25ºC, I=0.1M, pH=2 12

Figure S1. Example of the absorbance temporal decay of methylparaben due to the oxidation reaction initiated by peroxynitrite. Experimental conditions: $[MP]_0=1.00\times10^{-5}$M; $[HOONO]_0=4.00\times10^{-5}$M; pH=2; I=0.1 M; T=25ºC

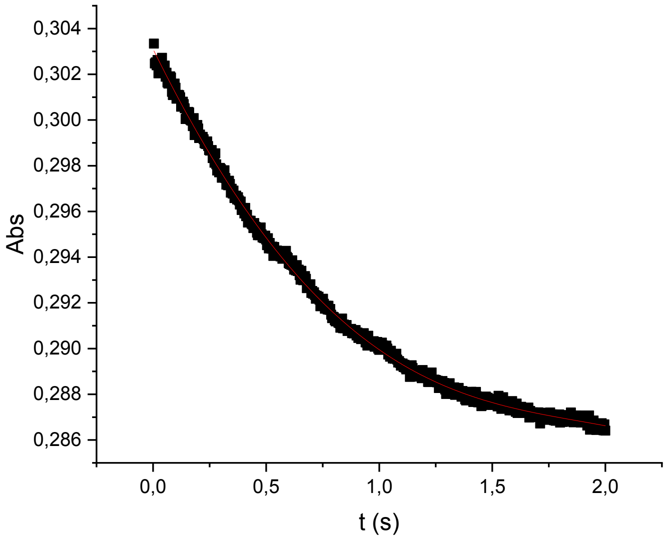

Table S1. Fitting parameters of the decay shown in Figure S1 for MP. Experimental Conditions: $[MP]_0=1.00\times10^{-5}$M; $[HOONO]_0=4.00\times10^{-5}$M; pH=2; I=0.1 M; T=25ºC

	Value	***Standard Error***		
Intercept	0,3031	3,95537E-5	**Number of Points**	500
Slope 1	-0,02009	2,72908E-4	**Degrees of Freedom**	495
Slope 2	0,00725	5,53011E-4	**Residual Sum of Squares**	1,51197E-5
Slope 3	3,77619E-5	4,14422E-4	**R-Square (COD)**	0,99862
Slope 4	-3,50816E-4	1,02591E-4	**Adj. R-Square**	0,99861

In this experiment:

Slope 1= -0.02009 s^{-1} as Ɛ= 15788 M^{-1} cm^{-1}, then v_0= 1.27×10^{-6} M s^{-1}

Table S2. Initial rates for the oxidation of the investigated parabens by peroxynitrite. Experimental conditions: pH=2, I=0.1M; T=25 ºC.

$v_0 \times 10^6$ (M/s)		$[MP]_0 \times 10^5$ (M)				
		1	2	4	6	8
$[HOONO]_0 \times 10^5$ (M)	4.0	1.86±0.01	2.24±0.01	2.68±0.02	3.07±0.02	3.51±0.01
	6.0	2.79±0.01	3.36±0.01	4.01±0.02	4.71±0.03	5.28±0.01
	8.0	3.66±0.01	4.37±0.01	5.28±0.02	5.98±0.01	6.55±0.02
	10.0	4.01±0.02	4.75±0.04	5.77±0.01	6.59±0.02	7.89±0.04
	12.0	5.12±0.03	5.85±0.01	6.71±0.05	7.85±0.01	9.01±0.03
$v_0 \times 10^6$ (M/s)		$[EP]_0 \times 10^5$ (M)				
		1	2	4	6	8
$[HOONO]_0 \times 10^5$ (M)	4.0	1.10±0.02	1.23±0.01	1.32±0.02	1.45±0.03	1.52±0.03
	6.0	1.62±0.01	1.76±0.01	1.95±0.01	2.06±0.03	2.23±0.01
	8.0	2.08±0.02	2.27±0.01	2.58±0.02	2.82±0.01	2.96±0.02
	10.0	2.61±0.02	2.81±0.02	3.10±0.01	3.38±0.01	3.61±0.01
	12.0	3.17±0.02	3.39±0.01	3.68±0.03	3.96±0.03	4.32±0.03
$v_0 \times 10^7$ (M/s)		$[PP]_0 \times 10^5$ (M)				
		1	2	4	6	8
$[HOONO]_0 \times 10^5$ (M)	4.0	2.28±0.6	2.98±0.4	3.53±0.4	4.06±0.6	4.87±0.2
	6.0	3.52±0.2	4.27±0.3	5.13±0.4	5.97±0.4	7.02±0.1
	8.0	4.52±0.3	5.78±0.5	7.14±0.5	8.54±0.3	9.76±0.4
	10.0	5.59±0.5	6.84±0.5	8.54±0.5	10.30±0.5	12.10±0.4
	12.0	7.37±0.1	8.64±0.1	10.30±0.3	12.34±0.3	14.20±0.3
$v_0 \times 10^7$ (M/s)		$[BP]_0 \times 10^5$ (M)				
		1	2	4	6	8
$[HOONO]_0 \times 10^5$ (M)	4.0	2.91±0.01	3.31±0.01	3.39±0.02	4.18±0.03	4.62±0.04
	6.0	4.08±0.01	4.72±0.01	5.26±0.02	5.98±0.05	6.73±0.01
	8.0	5.32±0.03	6.35±0.01	7.05±0.02	8.33±0.01	9.52±0.02
	10.0	6.83±0.02	7.76±0.02	9.03±0.01	10.40±0.01	11.60±0.03
	12.0	8.23±0.04	9.10±0.01	10.90±0.02	12.40±0.01	13.70±0.01

Figure S2. Influence of $[MP]_0$ (left) and $[HOONO]_0$ (right) upon the initial rate (v_0). Experimental conditions: pH 2, I=0.1M and T=25ºC

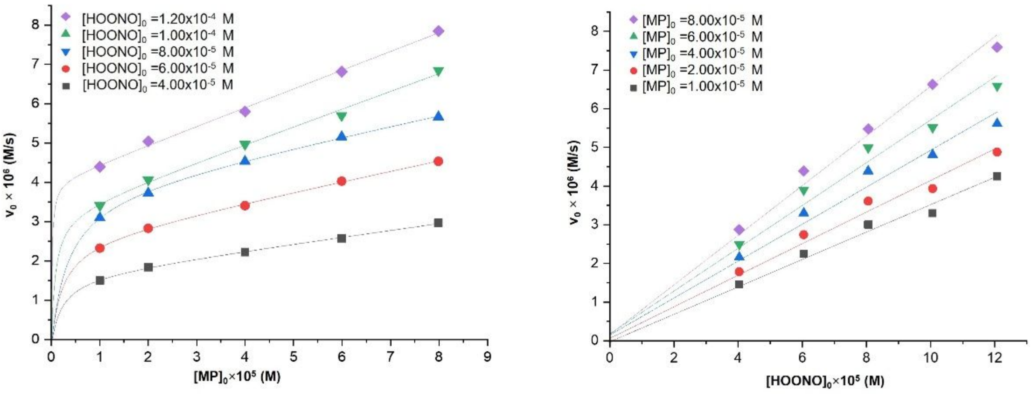

Figure S3. Influence of $[EP]_0$ (left) and $[HOONO]_0$ (right) upon the initial rate (v_0). Experimental conditions: pH 2, I=0.1M and T=25ºC

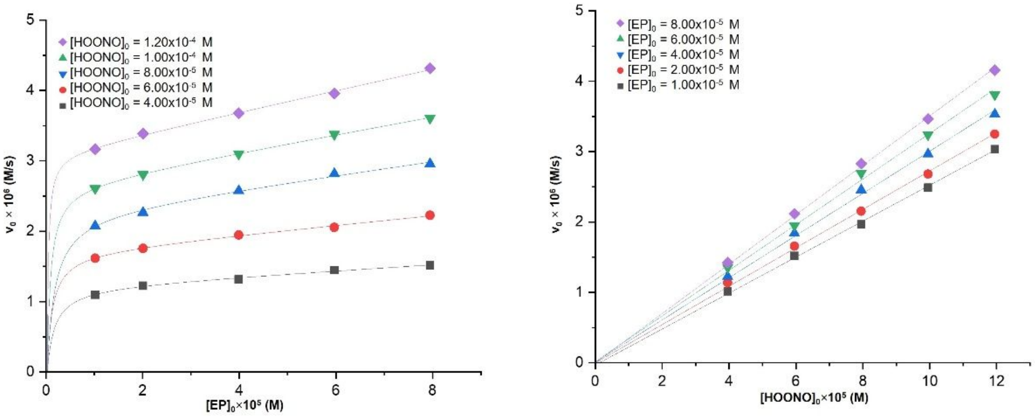

Figure S4. Influence of $[PP]_0$ (left) and $[HOONO]_0$ (right) upon the initial rate (v_0). Experimental conditions: pH 2, I=0.1M and T=25ºC

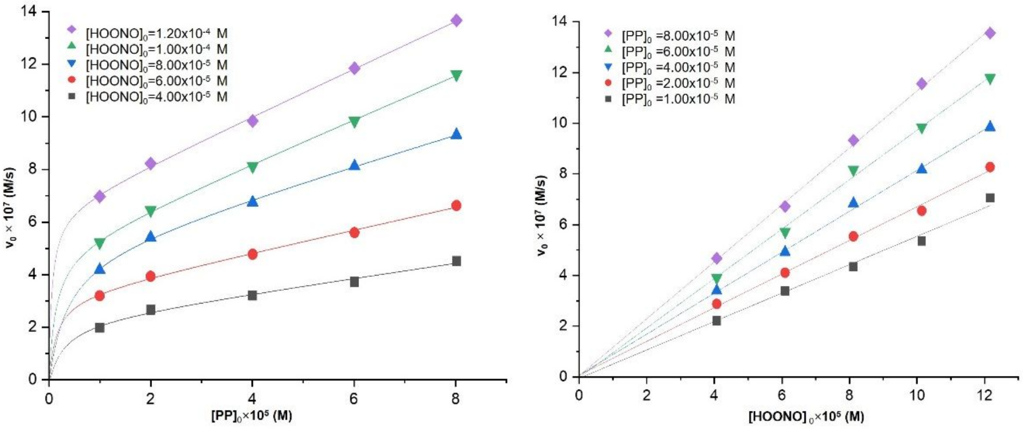

Figure S5. Influence of $[BP]_0$ (left) and $[HOONO]_0$ (right) upon the initial rate (v_0). Experimental conditions: pH 2, I=0.1M and T=25ºC

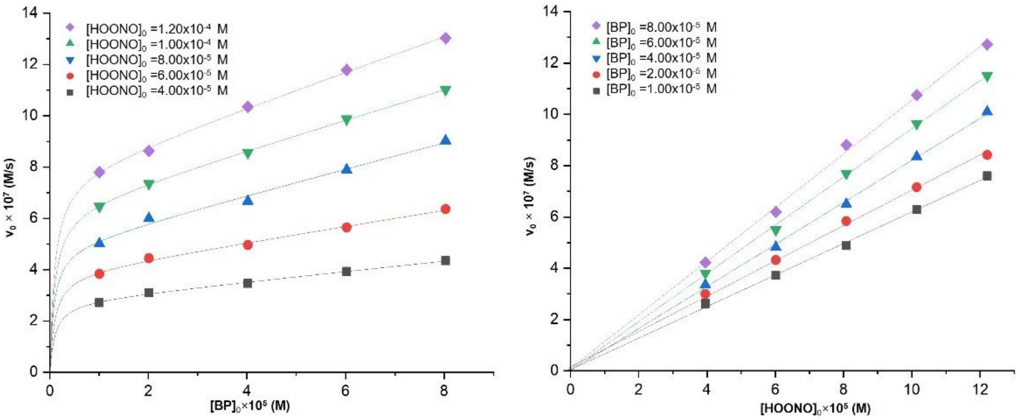

Figure S6. Influence of $[H^+]$ upon the initial rate (v_0) for all the parabens. Experimental conditions: $[Paraben]_0$=4.00×10^{-5} M, $[HOONO]_0$=8.00×10^{-5}M, I=0.1M, T^a=25ºC.

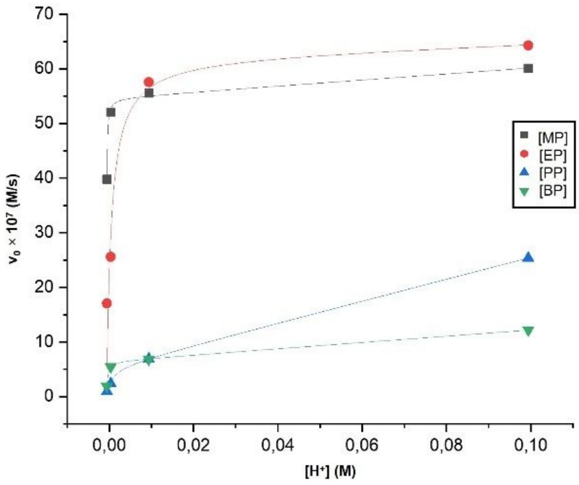

Table S3: Initial velocity values obtained at different ionic strength values $[Paraben]_0$=4.00×10^{-5} M, $[HOONO]_0$=8.00×10^{-5}M, pH = 2, T=25ºC.

**MP (Methylparaben)**					
**I (M)**	0.3	0.2	0.1	0.05	0.025
**v_o×10^6 (M/s)**	5.28±0.03	5.30±0.01	5.28±0.02	5.26±0.02	5.25±0.02
**EP (Ethylparaben)**					
**I (M)**	0.3	0.2	0.1	0.05	0.025
**v_o×10^6 (M/s)**	2.52±0.01	2.76±0.03	2.58±0.02	2.61±0.03	2.49±0.02
**PP (Propylparaben)**					
**I (M)**	0.3	0.2	0.1	0.05	0.025
**v_o×10^7 (M/s)**	7.29±0.03	8.10±0.01	7.14±0.02	8.84±0.02	7.17±0.02
**BP (Butylparaben)**					

I (M)	0.3	0.2	0.1	0.05	0.025
$v_o \times 10^7$ (M/s)	7.13±0.01	7.39±0.03	7.05±0.02	6.95±0.03	6.08±0.02

Table S4. Influence of the addition of nitrates and nitrites on the initial reaction rate. Experimental conditions: $[HOONO]_0 = 8.00\times10^{-5}$ M, $[MP]_0 = 4.00\times10^{-5}$ M, I= 0.1M, pH = 2, T= 25ºC.

Concentrations	$[NO_2^-]$ (M)		$[NO_3^-]$ (M)	
	0	8.00×10^{-5}	0	8.00×10^{-5}
$v_0 \times 10^6$ ($M\cdot s^{-1}$)	5.28± 0.01	5.27± 0.02	5.28± 0.01	5.25± 0.01

Table S5. Influence of free radical Scavenger (Acrylic Acid) on the initial reaction rate. Experimental conditions: $[HOONO]_0 = 8.00\times10^{-5}$ M, $[Paraben]_0 = 4.00\times10^{-5}$ M, I= 0.1M, pH = 2, T= 25ºC.

[Acrylic Acid] $\times10^5$ (M)	0.00	1.00	1.60	2.50	3.50	4.00	6.00	8.00
$v_{0MP} \times10^6$ (M/s)	5.28±0.02	4.73±0.01	3.77±0.02	3.01±0.07	2.41±0.01	1.79±0.02	1.34±0.01	1.39±0.04
$v_{0EP} \times10^6$ (M/s)	2.58±0.02	2.22±0.02	1.61±0.01	1.14±0.20	0.80±0.02	0.49±0.01	0.52±0.02	0.59±0.03
$v_{0PP} \times10^6$ (M/s)	0.71±0.50	0.59±0.03	0.41±0.30	0.26±0.02	0.16±0.01	0.04±0.08	0.05±0.05	0.05±0.02
$v_{0BP} \times10^6$ (M/s)	0.71±0.02	0.60±0.02	0.41±0.05	0.26±0.02	0.15±0.01	0.06±0.04	0.06±0.02	0.07±0.02

Table S6. Influence of temperature on the initial rate Experimental conditions: $[HOONO]_0 = 8.00\times10^{-5}$ M, $[Paraben]_0 = 4.00\times10^{-5}$ M, I= 0.1M, pH = 2.

MP	Temperature (K)	283	288	293	298	303
Methylparaben	$v_0\times10^6$ ($M\cdot s^{-1}$)	2.00±0.02	2.54±0.02	3.27±0.01	5.28±0.02	7.02±0.02
EP	Temperature (K)	283	288	293	298	303
Ethylparaben	$v_0\times10^6$ ($M\cdot s^{-1}$)	0.73±0.01	0.82±0.02	1.42±0.02	2.58±0.03	4.81±0.02
PP	Temperature (K)	283	288	293	298	303
Propylparaben	$v_0\times10^7$ ($M\cdot s^{-1}$)	0.73±0.02	3.22±0.01	4.42±0.02	7.14±0.02	22.1±0.04
BP	Temperature (K)	283	288	293	298	303
Butylparaben	$v_0\times10^7$ ($M\cdot s^{-1}$)	0.80±0.03	4.35±0.02	6.08±0.01	7.05±0.02	8.69±0.01

Deduction of the theoretical rate equation according with Scheme 1 of the article.

Theoretical rate equation

$$v_0 = \left(-\frac{d[RP]}{dt}\right) = 2k_2[Paraben^+][Rad] + k_4[Paraben^+][HOONO] + k_6[X][Paraben^+]$$

Take in account a mass balance equation for the peroxynitrite concentration.

$$[HOONO]_0 = [HOONO] + [ONOO^-]$$

$$[HOONO] = [HOONO]_0\left(\frac{[H^+]}{[H^+] + K_A}\right)$$

$$A' = \left(\frac{[H^+]}{[H^+] + K_A}\right)$$

$$[HOONO] = [HOONO]_0 A'$$

If the equilibrium 2 is considered it can write:

$$[Paraben^{+}] = \frac{[\mathrm{Paraben}]_0[\mathrm{H}^{+}]}{\mathrm{K}_{\mathrm{A'}}}$$

Applying the steady-state approximation to [X]

$$\left(\frac{d[X]}{dt}\right) = 0 = k_4[Paraben^{+}][HOONO] - k_5[X] - k_6[X][Paraben^{+}]$$

$$[X] = \left(\frac{k_4[Paraben^{+}][HOONO]}{k_5 + k_6[Paraben^{+}]}\right)$$

Substitute [HOONO] and [Paraben^{+}]:

$$[X] = \left(\frac{k_4[Paraben]_0[H^{+}][HOONO]_0 A'}{k_5 K_B + k_6[Paraben]_0[H^{+}]}\right)$$

Substituting all concentration expressions on the mass balance for peroxynitrite, taking into account the equilibria deduced in the reaction mechanism.

$$v_0 = \left(\frac{2k_2[Paraben]_0[H^{+}][Rad]}{K_{A'}}\right) + \left(\frac{k_4 k_5 K_{A'}[Paraben]_0[H^{+}][HOONO]_0 A' + 2k_6 k_4[Paraben]_0{}^2[H^{+}]^2[HONOO]_0 A'}{k_5 K_{A'}^2 + k_6 K_{A'}[Paraben]_0[H^{+}]}\right)$$

Applying the steady-state approximation to [Rad]

$$\frac{d[Rad]}{dt} = 0 = k_1[HOONO] - k_2[Paraben^{+}][Rad]$$

$$[Rad] = \frac{K_{A'} k_1[HOONO]_0 A'}{k_2[Paraben]_0[H^{+}]}$$

Substituting [Rad] on the theoretical rate equation

$$v_0 = \left(\frac{2k_1 k_5 K_{A'}^2[HOONO]_0 A' + 2k_1 k_6 K_{A'}[HOONO]_0 A' + k_4 k_5 K_{A'}[Paraben]_0[H^{+}][HOONO]_0 A' + 2k_6 k_4[Paraben]_0{}^2[H^{+}]^2[HONOO]_0 A'}{k_5 K_{A'}^2 + k_6 K_{A'}[Paraben]_0[H^{+}]}\right)$$

Bring out common factor $[HOONO]_0$ A'

$$v_0 = \left(\frac{2k_1 k_5 K_{A'}^2 + 2k_1 k_6 K_{A'} + k_4 k_5 K_{A'}[Paraben]_0[H^{+}] + 2k_6 k_4[Paraben]_0{}^2[H^{+}]^2}{k_5 K_{A'}^2 + k_6 K_{A'}[Paraben]_0[H^{+}]}\right)[HOONO]_0 A'$$

The only interest rate constant that can be calculated is k_4.

$$[HOONO]_0 = \frac{A[Paraben]_0 + B[Paraben]_0{}^2}{1 + C[Paraben]_0}$$

$$[Paraben]_0 = D[HOONO]_0$$

Table S7. Values of the parameters A, B, C and D form the adjust of the representation of initial rate vs substrate or oxidant. Experimental Conditions: T=25°C, pH=2, I=0.1M.

**Methylparaben**

$[HOONO]_0 \times 10^5$ (M)	$A \times 10^1$ (M/s^3)	$B \times 10^{-4}$ (M/s^2)	$C \times 10^{-5}$ (s^{-2})	r
4.00	9.95±0.04	0.91±0.01	4.81±0.03	0,99978
6.00	14.5±0.01	1.35±0.02	4.64±0.02	0,9998
8.00	13.8±0.02	0.86±0.06	3.03±0.01	0,99988
10.0	50.1±0.05	6.53±0.03	13.0±0.04	0,99863
12.0	306.0±0.03	34.8±0.04	65.2±0.01	0,99937
$[MP]_0 \times 10^5$ M	D (M/s)		r	
1.00	0.41±0.03		0,98971	
2.00	0.47±0.02		0,98564	
4.00	0.56±0.03		0,9804	
6.00	0.64±0.01		0,98272	
8.00	0.75±0.06		0,99087	
**Ethylparaben**				
$[HOONO]_0 \times 10^5$ (M)	$A \times 10^1$ (M/s^3)	$B \times 10^{-4}$ (M/s^2)	$C \times 10^{-5}$ (s^{-2})	r
4.00	8.23±0.04	0.27±0.01	6.71±0.03	0,9995
6.00	16.6±0.01	0.63±0.02	9.65±0.02	0,99977
8.00	14.8±0.02	0.60±0.06	6.44±0.01	0,99944
10.0	38.4±0.05	1.77±0.03	14.4±0.04	0,99996
12.0	126.0±0.03	6.11±0.04	40.6±0.01	0,99981
$[EP]_0 \times 10^5$ M	D (M/s)		r	
1.00	0.26±0.03		0,99933	
2.00	0.27±0.06		0,99876	
4.00	0.30±0.03		0,99818	
6.00	0.33±0.01		0,99755	
8.00	0.35±0.02		0,99933	
**Propylparaben**				
$[HOONO]_0 \times 10^5$ (M)	$A \times 10^1$ (M/s^3)	$B \times 10^{-4}$ (M/s^2)	$C \times 10^{-5}$ (s^{-2})	r
4.00	9.99±0.04	1.06±0.01	3.74±0.03	0,997
6.00	26.81±0.01	3.24±0.02	7.46±0.02	0,99923
8.00	16.35±0.02	1.73±0.06	2.97±0.01	0,99977
10.0	42.71±0.05	6.55±0.03	7.76±0.04	0,99983
12.0	119.8±0.03	15.93±0.04	17.32±0.01	0,99965
$[PP]_0 \times 10^5$ M	D (M/s)		r	
1.00	0.59±0.03		0,99688	
2.00	0.70±0.06		0,99882	
4.00	0.85±0.03		0,99949	
6.00	1.03±0.01		0,99933	
8.00	1.19±0.02		0,99959	
**Butylparaben**				
$[HOONO]_0 \times 10^5$ (M)	$A \times 10^1$ (M/s^3)	$B \times 10^{-4}$ (M/s^2)	$C \times 10^{-5}$ (s^{-2})	r
4.00	31.41±0.04	2.18±0.01	10.48±0.03	0,99958
6.00	44.68±0.01	3.44±0.02	10.69±0.02	0,99912
8.00	68.32±0.02	6.87±0.06	12.93±0.01	0,99787
10.0	75.53±0.05	6.74±0.03	11.02±0.04	0,99992
12.0	90.67±0.03	7.88±0.04	11.03±0.01	0,9997
$[BP]_0 \times 10^5$ M	D (M/s)		r	

1.00	0.67±0.03	0,99881
2.00	0.75±0.06	0,99852
4.00	0.90±0.03	0,99922
6.00	1.03±0.01	0,99943
8.00	1.15±0.02	0,99882

Table S8. Parameters A, B and C obtained from the fit of the oxidant concentration versus A, B and C parameters from Tables S7. Experimental conditions: I=0.1 M; T=25ºC; pH=2.

Parabens	A×10^{-5} (M^2/s^3)	B×10^{-9} (M^2/s^2)	C ×10^{-9} (M/s^2)
**MP**	3.14±0.13	3.65±0.27	64.5±0.04
**EP**	1.29±0.32	0.64±0.12	36.3±0.09
**PP**	1.18±0.25	1.65±0.09	13.7±0.73
**BP**	0.74±0.04	0.73±0.19	0.72±0.03

Considering:

$$v_0 = \left(\frac{2k_1k_5K_{A'}^2 + 2k_1k_6K_{A'}[Paraben]_0[H^+] + k_4k_5K_{A'}[Paraben]_0[H^+] + 2k_6k_4[Paraben]_0{}^2[H^+]^2}{k_5K_{A'}^2 + k_6K_{A'}[Paraben]_0[H^+]}\right)[HOONO]_0A'$$

And like:

$$[HOONO]_0 = \frac{A[Paraben]_0 + B[Paraben]_0{}^2}{1 + C[Paraben]_0}$$

$$v_0 = \left(\frac{2\,k_1 + \left(\frac{2k_1k_6[Paraben]_0[H^+]}{k_5K_{A'}}\right) + \left(\frac{k_4[Paraben]_0[H^+]}{K_{A'}}\right) + \left(\frac{2k_6k_4[Paraben]_0{}^2[H^+]^2}{k_5K_{A'}^2}\right)}{1 + \left(\frac{k_6[Paraben]_0[H^+]}{k_5K_{A'}}\right)}\right)[HOONO]_0A'$$

So:

$$A = \left(\frac{k_4[Paraben]_0[H^+]}{K_{A'}}\right) or \left(\frac{2k_1k_6[Paraben]_0[H^+]}{k_5K_{A'}}\right)$$

$$B = \left(\frac{2k_6k_4[Paraben]_0{}^2[H^+]^2}{k_5K_{A'}^2}\right)$$

$$C = \left(\frac{k_6[Paraben]_0[H^+]}{k_5K_{A'}}\right)$$

$K_{A'}$ determination

Table S9. Initial rate of the different parabens at different pH. Experimental conditions: [Paraben]$_0$=4.00×10^{-5} M; [HOONO]$_0$=8.00×10^{-5}M, T=25°C.

pH	V_{0MP}×10^7 (M/s)	V_{0EP}×10^7 (M/s)	V_{0PP}×10^7 (M/s)	V_{0BP}×10^7 (M/s)
**3**	0.02±0.01	0.29±0.01	0.06±0.01	0.20±0.01
**2.5**	0.01±0.01	0.31±0.03	0.04±0.02	0.20±0.01
**2**	0.01±0.01	0.31±0.02	0.03±0.01	1.15±0.01
**1.5**	0.16±0.04	1.26±0.03	1.02±0.02	1.79±0.03
**1**	1.01±0.02	4.26±0.27	1.84±0.01	2.40±0.02
**0.5**	6.20±0.03	1.12±0.12	1.38±0.01	9.29±0.10
**0**	9.96±0.05	3.99±0.09	14.3±0.09	15.3±0.18
**-0.5**	4.36±0.25	42.4±0.19	45.1±0.24	144.4±0.34
**-1**	246.4±0.32	734.1±0.87	494.9±0.43	812.2±0.52

Table S10. Initial rate of the different parabens and h_0. Experimental conditions: $[Paraben]_0=4.00\times10^{-5}$ M; $[HOONO]_0=8.00\times10^{-5}$M, T=25ºC.

H_0[1]	$V_0/[MP]_0 \times10^3$ (s^{-1})	$V_0/[EP]_0 \times10^2$ (s^{-1})	$V_0/[PP]_0 \times10^2$ (s^{-1})	$V_0/[BP]_0 \times10^2$ (s^{-1})
**0.26302998**	0.050±0.01	0.074±0.01	0.015±0.01	0.052±0.01
**0.26343003**	0.043±0.01	0.078±0.03	0.012±0.02	0.050±0.01
**0.26469912**	0.038±0.01	0.079±0.02	0.009±0.01	0.28±0.01
**0.26875271**	0.41±0.04	0.31±0.03	0.25±0.02	0.44±0.03
**0.28198435**	2.54±0.02	1.06±0.27	0.46±0.01	0.60±0.02
**0.32826798**	15.5±0.03	0.28±0.12	0.34±0.01	2.32±0.10
**0.53082333**	24.9±0.05	0.99±0.09	3.58±0.09	3.84±0.18
**2.42652771**	10.9±0.25	10.6±0.19	11.2±0.24	36.13±0.34
**296.619706**	616.1±0.32	183.6±0.87	123.7±0.43	203.1±0.52

[1]. Coussemant, F., Hellin, M.,Torck, B. Les fonctions d'acidité et leurs utilisations en catalyse acido-basique, Gordon et Breach, Paris Londres New-York (1969)

Considering this mechanism (figure S7) and the adjust (E9) can be determinate the parameter k and K_B

Figure S7. Singman mechanism for ester hydrolysis

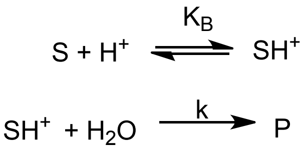

Table S11. Parameters k and K_B from the representation of table S10. Experimental conditions: $[Paraben]_0=4.00\times10^{-5}$ M; $[HOONO]_0=8.00\times10^{-5}$M, T=25ºC.

Parabens	$k\times10^3$ (M^{-1} s^{-1})	$K_B\times10^2$ (M^{-1})
**MP**	0.127±0.01	0.812±0.01
**EP**	0.753±0.02	1.942±0.01
**PP**	0.859±0.03	3.516±0.02
**BP**	2.77±0.01	7.234±0.01

k_4 determination

For example, k_4 for MP was calculated as:

Knowing that:

$[H^+]=10^{-2}$ M
$K_A=10^{-6,5}$
$A'=([H+]/[H+]+K_A)$
$K_{A'}=1/K_B$

For methylparaben:

$$A=\left(\frac{k_4[Paraben]_0[H^+]}{K_{A'}}\right)[HOONO]_0A'$$

$$A'=\frac{10^{-2}}{10^{-2}+10^{-6,5}}=0,99996$$

$$A = \left(\frac{k_4[H^+]A'}{K_{A'}}\right)$$

$$3142493{,}75 = \frac{k_4 10^{-2} 0{,}99996}{123{,}1527}$$

$$\boldsymbol{k_4 = 3{,}87\ 10^{10}\ M^{-1}s^{-1}}$$

Figure S8. Linear fits of natural logarithm of k_4 and $K_{A'}$ plotted against the steric constant (A) and the inductive constant (B)

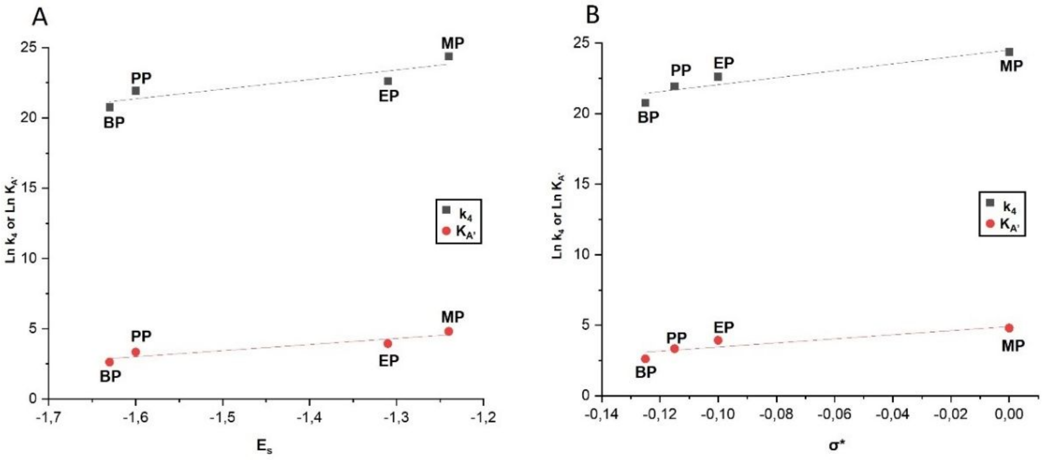

Table S12. Summarizes the values of (ln k_4), (ln $K_{A'}$) and steric and electronic constants for each paraben.

	ln k_4	ln $K_{A'}$	σ* [2]	E_s [3]
**(MP) -CH_3**	22.07±0.01	4.81±0.01	0	-1,24
**(EP) -C_2H_5**	20.31±0.02	3.94±0.01	-0,1	-1,31
**(PP) -C_3H_7**	19.63±0.01	3.34±0.02	-0,115	-1,6
**(BP) -C_4H_9**	18.45±0.03	2.62±0.01	-0,125	-1,63

[2]. Pal'm, V. A. (1961). Structure and reactivity of organic compounds (quantitative laws). Russian Chemical Reviews, 30(9), 471. DOI 10.1070/RC1961v030n09ABEH002997

[3]. Hansch, C., Rockwell, S. D., Jow, P. Y., Leo, A., & Steller, E. E. (1977). Substituent constants for correlation analysis. Journal of medicinal chemistry, 20(2), 304-306. https://doi.org/10.1021/jm00212a024

Figure S9. HPLC analysis spectra for acid media. Experimental conditions: [Paraben]$_0$=8.00×10^{-5} M; [HOONO]$_0$=12.00×10^{-5}M, T=25ºC, I=0.1M, pH=2

RT = 3.19 min Hydroquinone

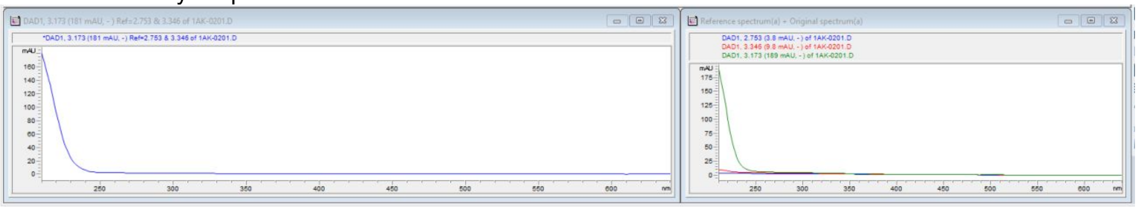

RT = 5.23 min Methylparaben

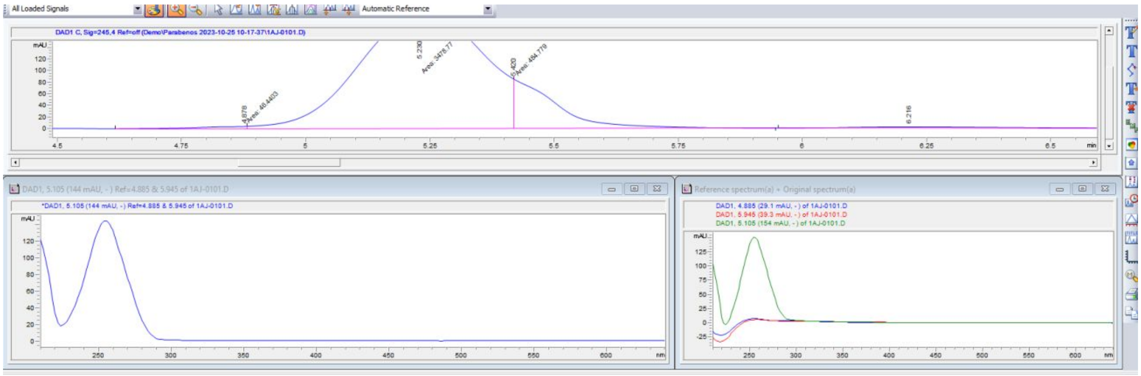

RT = 5.42 min p-Hydroxybenzoic Acid

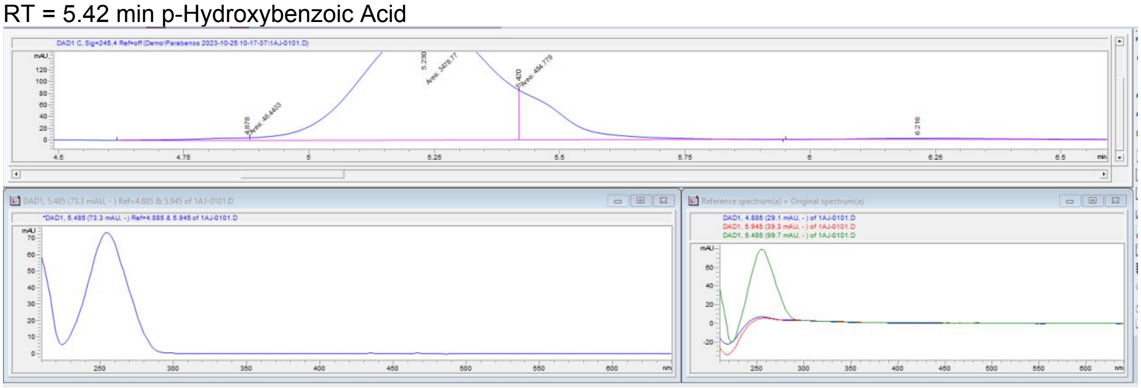

RT = 6.21 min Quinone

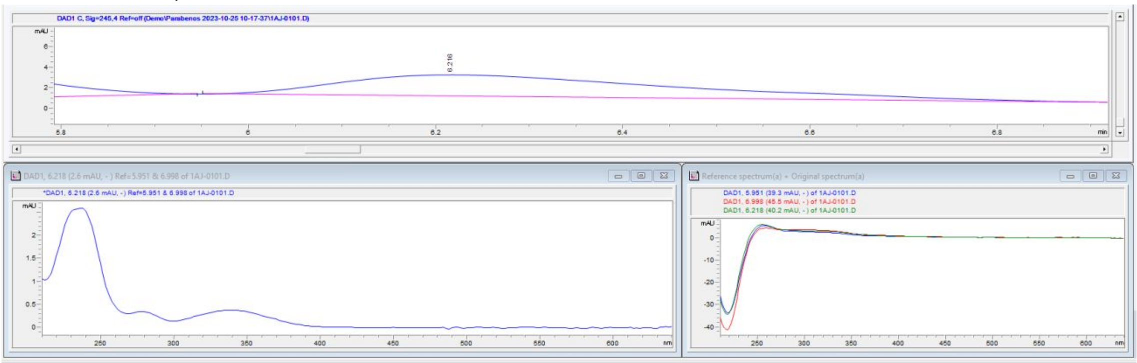

Supplement: Supplementary file 1 [file ew5c00362_si_001.pdf]
